# Dual-targeted repetitive transcranial magnetic stimulation modulates brain functional network connectivity to improve cognition in mild cognitive impairment patients

**DOI:** 10.3389/fphys.2022.1066290

**Published:** 2022-11-18

**Authors:** Xinqi Zhang, Huixia Ren, Zian Pei, Chongyuan Lian, XiaoLin Su, Xiaoyong Lan, Chanjuan Chen, YuHua Lei, Baima Li, Yi Guo

**Affiliations:** ^1^ Department of Neurology, Shenzhen People’s Hospital, The Second Clinical Medical College of Jinan University, The First Affiliated Hospital of Southern University of Science and Technology, Shenzhen, China; ^2^ Department of Geriatrics, Shenzhen People’s Hospital (The Second Clinical Medical College of Jinan University, The First Affiliated Hospital of Southern University of Science and Technology), Shenzhen, China; ^3^ Shenzhen Bay Laboratory, Institute of Neurological Disease, Shenzhen, China

**Keywords:** mild cognitive impairment (MCI), repetitive transcranial magnetic stimulation (rTMS), intervention, default mode network (DMN), memory, cognitive function

## Abstract

**Background:** Mild cognitive impairment (MCI) is a condition between normal aging and dementia; nearly 10–15% of MCI patients develop dementia annually. There are no effective interventions for MCI progression. Repetitive transcranial magnetic stimulation (rTMS) is a non-invasive brain stimulation technique that has attempted to improve the overall cognitive function of MCI patients. However, it does not affect episodic memory improvement.

**Methods:** In this study, we engaged 15 clinically diagnosed MCI patients and normal controls to explore the effect of dual-targeted rTMS on progressing cognitive function, particularly episodic memory in MCI patients. Resting-state EEG recordings and neuropsychological assessments were conducted before and after the intervention. EEG features were extracted using an adaptive algorithm to calculate functional connectivity alterations in relevant brain regions and the mechanisms of altered brain functional networks in response to dual-target rTMS.

**Results:** The study revealed that the functional brain connectivity between the right posterior cingulate gyrus (PCC) and the right dorsal caudate nucleus (DC) was significantly reduced in MCI patients compared to normal controls (*p* < 0.001). Dual-target rTMS increased the strength of the reduced functional connectivity (*p* < 0.001), which was related to cognitive enhancement (*p* < 0.05).

**Conclusion:** This study provides a new stimulation protocol for rTMS intervention. Improving the functional connectivity of the right PCC to the right DC is a possible mechanism by which rTMS improves overall cognitive and memory function in MCI patients.

## Introduction

Mild cognitive impairment (MCI) is a transitional state between normal aging and dementia. MCI shows a progressive impairment of memory or other cognitive functions that are temporarily ineffective to the individual’s daily performance. MCI is prevalent in the elderly, with a prevalence of 16–20% in people aged 65 years and older (Petersen, 2004; Roberts and Knopman, 2013), and approximately 10–15% of people with MCI develop dementia yearly (Mitchell and Shiri-Feshki,2009; Roberts and Knopman, 2013). MCI is a considerable public health problem due to its prevalence, risk of progression to dementia, and lack of effective treatments.

Over the past decade, clinical drug trials had a 99.6% failure rate in treating Alzheimer’s disease (AD) (Cummings, et al., 2014; Schneider, et al., 2014). Current research suggests that pathological changes in the brain begin years before the onset of real cognitive impairment symptoms and may be irreversible by the time dementia is diagnosed (Sperling, et al., 2013). Therefore, researchers have shifted their attention to MCI, anticipating to intervene and limiting the progression of dementia at this stage. Certain economic analyses suggest that delaying the onset of dementia by 5 years might reduce the cost of treatment for the subsequent 2 decades of dementia by 40% (Alzheimer’s,2015). Clinical studies investigating whether cholinesterase inhibitors can slow the rate of transition to dementia in MCI or at least temporarily improve cognitive performance have yielded disappointing results (Doody, et al., 2009). The adverse effects of cholinesterase inhibitors are more evident than the improvement in cognitive function (Cooper, et al., 2013; Tricco, et al., 2013; Fitzpatrick-Lewis, et al., 2015). Aducanumab, an expensive drug recently approved by the FDA, is restricted to treating Aβ protein-positive MCI patients. The efficacy and safety of the treatment have also been widely questioned (Haeberlein, et al., 2020). Currently, a healthy lifestyle, particularly exercise, cardiovascular risk factor control, and cognitive/social activities, are the recommended approaches to deal with MCI. Apart from general health recommendations, there are no effective treatments for MCI.

Non-invasive-brain stimulation techniques have attracted much attention recently. Transcranial magnetic stimulation technology delivers solid magnetic pulses to the brain without attenuation through the skull to non-invasively stimulate and modulate the cerebral cortex (Ni and Chen, 2015; Rabey and Dobronevsky, 2016). Repetitive transcranial magnetic stimulation (rTMS) can regulate brain function by using multiple pulses emitted continuously at different frequencies to selectively activate or inhibit cortical excitability in both directions (Huang, et al., 2005). According to neuropathological, neurophysiological, and neuroimaging studies, there is a “functional disconnection” in the brain during the MCI phase (Rossini, et al., 2007; Maestú, et al., 2019). Researchers believe the brain’s impaired and altered functional connectivity is part of the cognitive structural compensation mechanism (Maestú, et al., 2019). During this process, cognitive skills can be protected by modulating the relevant brain regions or neural networks. A meta-analysis on the use of rTMS to enhance cognitive function in AD and MCI patients suggests that rTMS augments overall cognitive function in dementia patients (Chou, et al., 2020). Improvements in cognitive function have been shown in various randomized controlled trials of rTMS intrusions in patients with AD dementia (Cotelli, et al., 2006; 2011, Ahmed, et al., 2012; Rabey, et al., 2013; Rutherford, et al., 2015; Wu, et al., 2015; Zhao, et al., 2017).

The ideal stimulation site for rTMS to enhance cognitive function is still being explored. Its efficacy is influenced by the stimulation protocol, with varying choices of stimulation sites leading to distinct intervention outcomes (Seeley, et al., 2007; Balderston, et al., 2020). In rTMS studies on improving cognitive function in MCI and AD, the most frequently used stimulation site is the dorsolateral prefrontal cortex (DLPFC). The DLPFC plays a key role in memory encoding, attention, executive function, cognitive control, and emotional regulation (Riedel, et al., 2010; Ni and Chen, 2015; Lesenskyj, et al., 2018; Padmanabhan, et al., 2019). The precuneus (PCu), located in the posterior part of the medial parietal cortex, is the default mode network (DMN) area that interacts with other brain networks more frequently and constitutes the hippocampus contextual memory network (Tovote, et al., 2015). The PCu is a crucial node for the episodic memory impairment observed in early AD (Tovote, et al., 2015). In a crossover trial of “prodromal AD,” rTMS at the left PCu site significantly enhanced memory as assessed by the Rey auditory verbal learning delayed recall score; however, there was no significant change in other cognitive function scores (Koch, et al., 2018). A meta-analysis on improving cognitive function by rTMS indicated that high-frequency, multi-site, and long-session rTMS was more effective in enhancing MCI cognitive function (Zhang, et al., 2021). Based on the characteristics of altered brain networks in MCI cognitive impairment, prefrontal and parietal regions stimulation may have a better overall cognitive improvement effect (Badhwar, et al., 2017).

We hypothesized that dual-target rTMS on the left DLPFC + left PCu might have a better effect on improving memory in MCI patients. To demonstrate this idea, we employed a dual-target rTMS protocol on a group of MCI patients, using the neurofunctional scale to assess the effect of dual-target rTMS on cognition in MCI patients and the impact of dual-target rTMS on functional brain network connectivity in MCI patients using EEG.

## Materials and methods

### Participants

This research was approved by the Ethics Committee of Shenzhen People’s Hospital, following the Declaration of Helsinki recommendations, and registered with the Chinese Clinical Trials Registry (ChiCTR1800019199). All MCI patients were recruited at the Neurology Clinic of Shenzhen People’s Hospital between March 2021–May 2022. They were informed about the study and had signed an informed consent form before participation. The inclusion criteria for MCI patients were as follows: age 55–75 years, right-handed, meeting American Academy of Neurology (AAN) diagnostic criteria for mild cognitive impairment (Petersen, et al., 2018), a Harkinski ischemia score of less than four, and a Montreal Cognitive Assessment (MoCA) score between 26 and 18.

Exclusion criteria included the following: 1) history of severe organ dysfunction, 2) history of psychiatric disorders such as major depression, generalized anxiety disorder, and schizophrenia, 3); contraindications to rTMS such as metal implants, 4) any type of dementia, and 5) use of medications that may affect the EEG within 1 month before enrollment. A normal, age-matched, right-handed population was enrolled as MCI patient controls. The exclusion criterion was the absence of cognitive impairment, and the remaining criteria were the same for MCI patients.

Each participant was assessed on a cognitive scale and an affective scale, and a specialist neurologist performed the assessments at baseline and within 24 h of the completion of all stimuli. The Hamilton Anxiety Scale (HAMA) and the Hamilton Depression Scale (HAMD) were used to assessing anxiety and depression status. Overall cognitive performance was evaluated using the MoCA.

A comprehensive cognitive assessment of MCI patients was also conducted. Episodic memory was evaluated using the Chinese version of the Auditory-Verbal Learning Test (CVLT); executive function was evaluated using the Trail Making Test (TMT); attention and working memory were determined by the Digit Span Test (DST). The Symbol Digit Modalities Test (SDMT) was used to evaluate attention and information processing skills. The study utilized and randomized the A/B versions of the MoCA, CAVLT, and Digital Span.

### Transcranial magnetic stimulation intervention

MagPro X100 (Magventure, Denmark) with a 92 mm diameter figure-of-eight coil was used for the rTMS intervention. The left DLPFC was located at the “F3” electrode of the EEG 10–20 system, and the PCu was positioned at the midpoint of the “CPz” and “Pz” of the EEG 10–20 system. Patients received rTMS treatment five times a week for 4 weeks. The left DLPFC was stimulated first, and then the operator moved the coil to the PCu to complete the remaining stimulation. The rTMS parameters were DLPFC site: 10 Hz frequency, 1 s stimulation time and 10 s interval, 120 repetitions, and 1200 pulses; PCu site: 10 Hz frequency, 1 s stimulation time and 10 s interval, 80 repetitions, and 800 pulses. The stimulus intensity was set to 120% of the resting motor threshold. The resting motor threshold is the minimum intensity required to produce motor-evoked potentials.

## Resting-state EEG

### Data acquisition

EEG signal acquisition was performed once at baseline and once within 24 h after the completion of the intervention. Resting-state EEG was acquired for 8 min in the closed-eye (EC) condition using a Brainmap DC amplifier (Brain Products, Munich, Germany) with a 64-channel EEG system. Participants were instructed to sit in a comfortable chair and remain relaxed during the EEG recording. A conductive gel was used to maintain the electrode impedance below 5 kΩ. A sampling rate of 5,000 Hz was used, and the reference electrode was set to FCz.

### Pre-processing

1) Downsampling to 250 Hz; 2) applying a zero-phase finite impulse response filter for bandpass filtering between 1 and 45 Hz and removing noise and harmonics at 50 Hz by trap filtering; (3) eliminating noticeable artifacts by visual inspection; 4) discarding corrupted channels and spherical interpolation of rejected channels; and 5) applying independent component analysis to remove residual artifacts, including blinking, ECG signals, and high-frequency persistent muscle artifacts.

### EEG source localization and connectivity analysis

The extracted EEG data were subjected to source-level feature operations and assigned to the spatial anatomical partitioning template using thedynamic imaging of coherent sources (DICS) algorithm from the Fieldtrip software package (Oostenveld, et al., 2011). The Brainnetome atlas partitioning template was used to partition the brain anatomically (Fan, et al., 2016). The activity of the cortical sources was reconstructed from the cephalic surface topography. The Brainnetome atlas included estimating the sources’ size, possible location, direction, and magnitudes. Spatial source distribution was determined usingDICS. EEG alpha bands were obtained using Cohen MX.'s “gedBounds” frequency adaptive clustering algorithm (Cohen, 2021), and brain functional network connections were calculated.

The functional connection strength of EEG signals is evaluated using the phase synchronization metric. The phase difference calculates the phase synchronization between EEG signals, and the degree of phase synchronization is considered using the phase locking value (PLV) (Lachaux, et al., 1999). Assuming that $\phi_{rel}\left(t\right)$ is the phase difference of X(t) and Y(t). The following equation evaluates the PLVs:

$${PLV}_{xy}=\left|\frac{1}{N}\overset{N}{\underset{n=1}{\sum }}e^{i\phi_{rel}\left(t_{n}\right)}\right|,$$ (1)

where N is the length (i.e., number of points) of $\phi_{rel}\left(t\right)$ .

PLV assesses the phase difference time series distribution in [0, 2π). A significant value of PLV indicates that the phase difference time series occupies a negligible portion of the unit circle [0, 2π). The value of PLV ranges from 0 to 1. The greater the value, the greater the degree of phase synchronization between the two signals. If PLV equals 1, the phase difference time series is constant over the whole time series range; if PLV equals 0, the phase difference is uniformly distributed in the range [0, 2π).

### Statistical analysis

IBM Statistical Package for the Social Sciences (SPSS; IBM Corp., Armonk, NY, United States) version 25.0 was used to conduct statistical analyses. Subjects’ baseline data and measures in background information were expressed as mean ± standard deviation ( $\bar{x}$ ± SD) if they followed a normal distribution, otherwise expressed as median ± quartiles (M ± OD), and count information was expressed as percentages (%). SPSS 20.0 was used to analyze the study data statistically.

Between-group comparisons of baseline data were performed using one-way ANOVA (one-way analysis of variance). rTMS stimulus effects were compared based on the difference in their improvement effects, defined as the difference between post-stimulus and pre-stimulus, using paired samples *t*-test if the data were normally distributed and the Mann-Whitney nonparametric test otherwise. Statistical effect values were expressed as Cohen’s d and their 95% confidence intervals (95% CI). All comparisons were based on two-tailed tests, and the level of statistical significance was set at *p* < 0.05.

## Results

### Clinical information in baseline

The demographic and clinical information of the participants is shown in Table 1. The mean age and education of the MCI group were 66.40 (±6.09) years and 13.13 (±2.99) years, respectively; the mean age and education (± standard deviation) of the NC group were 63.75 (±2.46) years and 12.12 (±2.94) years, respectively. Age, gender, and education were not significantly different between the MCI and the control groups (all *p* > 0.05). The MCI group had lower MoCA scores than normal controls.

**TABLE 1:** Demographic and neuropsychological test results of MCI patients and normal controls.

Variable	MCI patients [*n* = 15, means (SD)]	Normal controls [*n* = 16, means (SD)]	χ^2^/t	*p*-value
Demographic				
Age	66.40 (6.09)	63.75 (2.46)	−1.569	0.134
Gender (M/F)	9/6	7/9	0.366	0.479
Education (years)	13.13 (2.99)	12.12 (2.94)	−0.945	0.352
Neuropsychological tests				
MoCA	21.93 (1.91)	27.62 (0.81)	10.696	< 0.001***
HAMA	4.60 (2.56)	1.75 (1.84)	−3.578	0.001**
HAMD	4.20 (3.57)	1.94 (2.32)	−2.11	0.044*

Abbreviations: M, male; F, female; MoCA, montreal cognitive assessment test; HAMA, hamilton anxiety rating scale; HAMD, hamilton depression rating scale. *p < 0.05; **p < 0.01; ***p < 0.001.

### Improved cognitive function in MCI patients after dual-targeted rTMS

**FIGURE 1:**
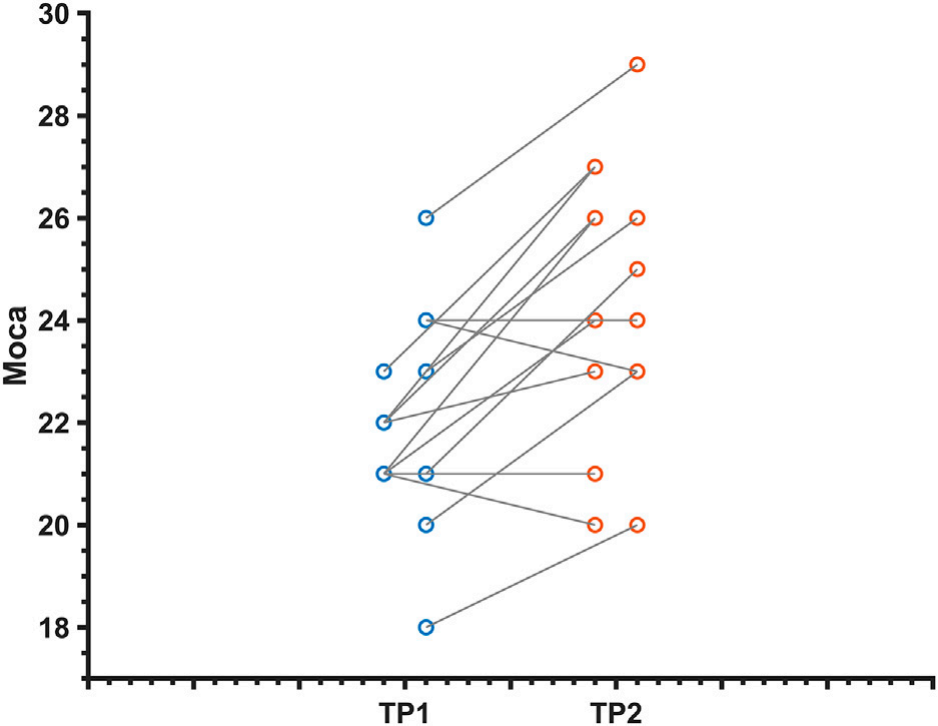
Change in MoCA scores in MCI patients before and after dual-target rTMS intervention.

Overall cognition and multiple cognitive functions were improved in MCI patients after dual-target rTMS compared to baseline levels (*p* < 0.05). The results showed that dual-target rTMS improved overall cognitive performance in MCI patients with MoCA (*T* = −4.39, *p* < 0.001), memory function CAVLT-immediately (*T* = −3.45, *p* < 0.01), CAVLT 5 min delay (*T* = −2.51, *p* < 0.05), CAVLT 20 min delay (*T* = −- 3.52, *p* < 0.01), executive function, connected test A (*T* = 2.45, *p* < 0.05), connected test B (*T* = 3.34, *p* < 0.01), and digital breadth backwards (*T* = −2.81, *p* < 0.05). See Table 2; Figure 1.

**TABLE 2:** Improvement in cognitive function after dual-target rTMS in MCI patients.

Variable	Pre [*n* = 15, means (SD)]	Post [*n* = 15, means (SD)]	% Of improvement	*t*-value	*p*-value
Neuropsychological tests					
MoCA	21.93 (1.91)	24.27 (2.66)	10.67%	−4.39	0.001**
Memory					
MoCA memory	1.00 (1.36)	1.93 (1.62)		−4.09	0.001**
CAVLT-immediately	7.27 (2.25)	8.60 (1.99)	18.29%	−3.45	0.004**
CAVLT-5 min	5.07 (3.17)	6.53 (3.70)	28.80%	−2.51	0.025*
CAVLT-20 min	4.66 (3.42)	6.40 (3.70)	37.33%	−3.52	0.003**
Executive function					
Trail Making A	79.46 (36.51)	66.06 (20.75)	16.86%	2.45	0.028*
Trail Making B	183.27 (66.05)	149.40 (46.96)	18.48%	3.34	0.005**
Attention					
digit span test (forward)	7.00 (1.41)	7.00 (1.07)	0	0.00	1.000
digit span test (backward)	4.13 (1.06)	4.73 (1.03)	14.53%	−2.81	0.014*
SDMT	30.40 (11.72)	32.93 (11.79)	8.32%	−1.53	0.149

Abbreviations: MoCA, montreal cognitive assessment test; CAVLT, the Chinese version of the Auditory Verbal Learning Test; SDMT, symbol digit modalities test. *p < 0.05; **p < 0.01; ***p < 0.001.

### Resting-state functional connectivity changes after dual-target rTMS

**FIGURE 2:**
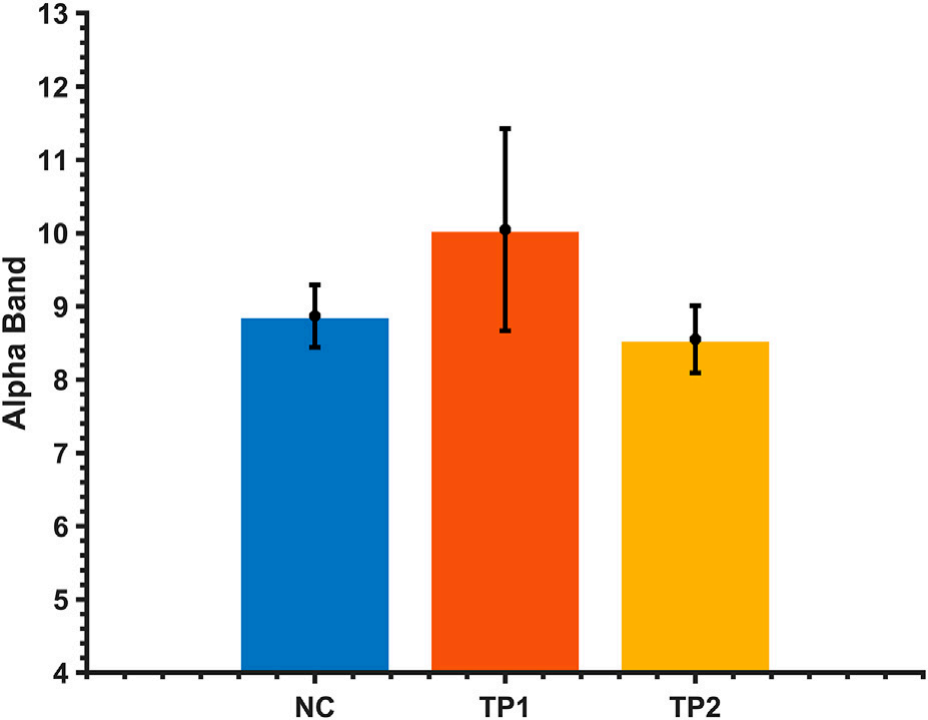
Comparison of the alpha average band range of MCI patients and normal controls before and after the dual-target rTMS intervention. The vertical axis is the frequency of the alpha band.

The alpha-band in the EEG data was extracted using an adaptive clustering method to calculate the network connectivity of all brain regions in the alpha-band. First, the alpha-band ranges of MCI patients were compared before and after rTMS intervention. No statistically significant difference was observed in the alpha-band (*p* > 0.05). Therefore, it can be concluded that the change in brain functional network connectivity in MCI patients before and after the intervention was not affected by the change of alpha-band before and after the intervention. See Figure 2.

The adaptive clustering method compared the alpha-band brain functional network connections in MCI patients before and after rTMS with normal controls. There were statistically significant variations between the brain functional network connections of MCI patients before and after rTMS and those of normal controls.

**FIGURE 3:**
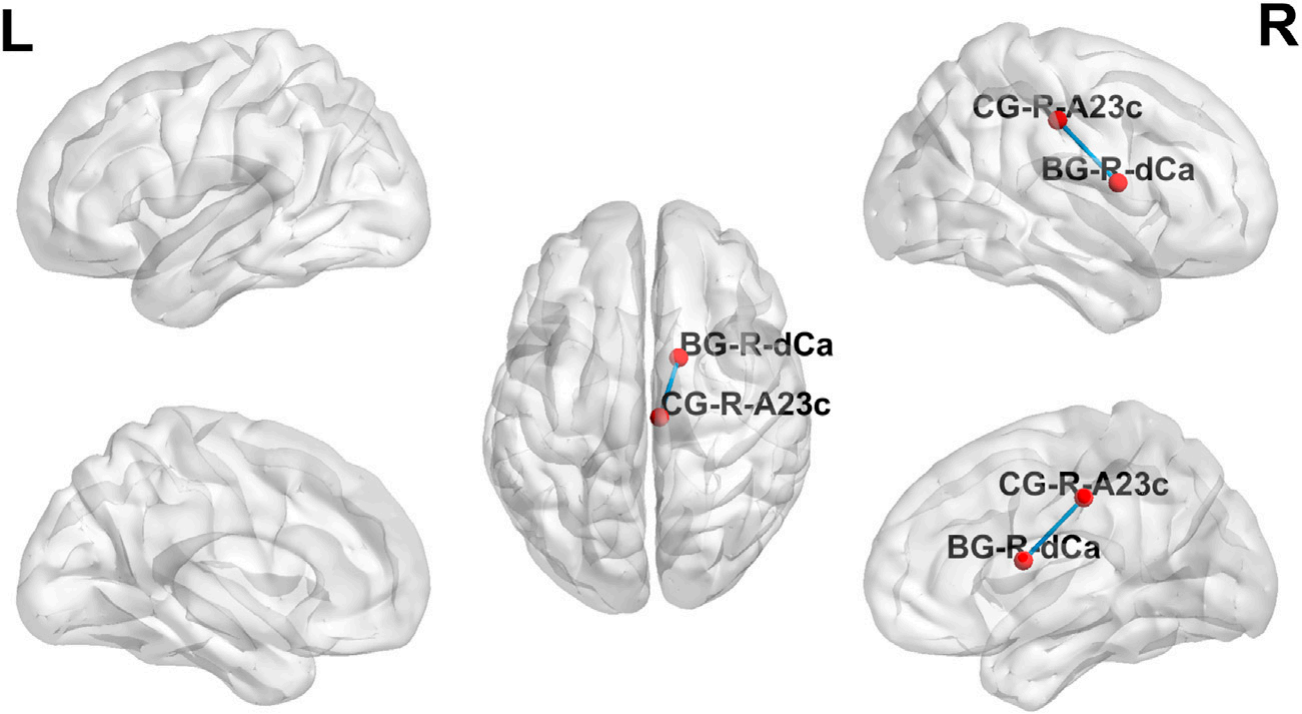
Brain functional network connectivity altered before and after dual-target rTMS intervention. BG, basal ganglia; CG, cingulate gyrus.

**FIGURE 4:**
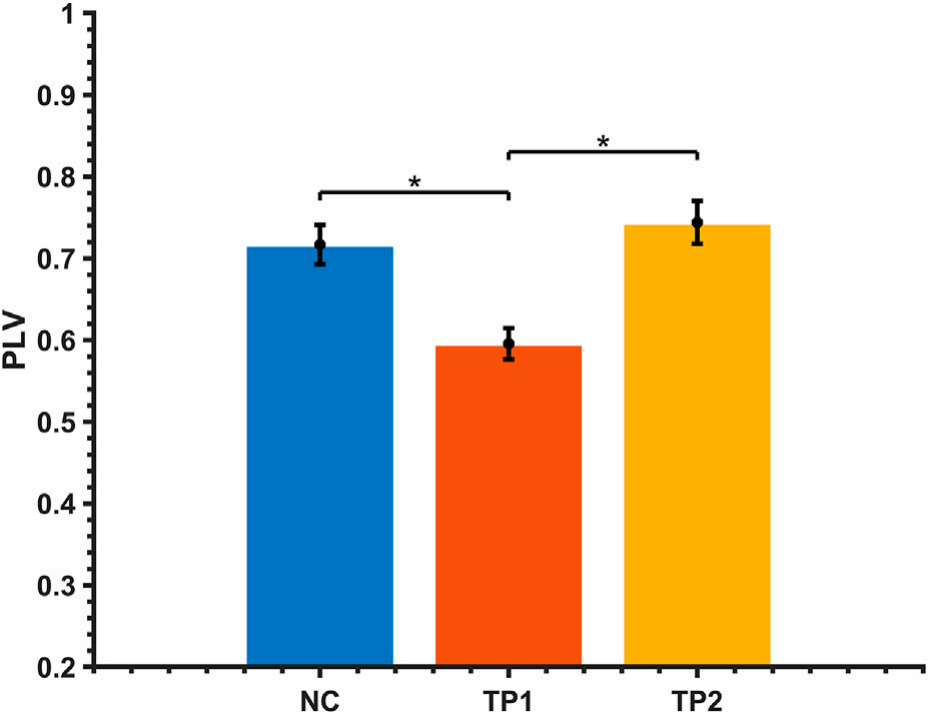
Comparison of alpha-band brain functional network connectivity in MCI patients before and after dual-target rTMS intervention with NC. *: *p* < 0.05.

The strength of the functional brain network connections between the right posterior cingulate gyrus (PCC) and the right dorsal caudate (DC) was altered in MCI patients after the dual-target rTMS intervention. The connection between the right PC and the right DC was weak before the rTMS intervention compared to normal controls (*p* < 0.001); however, the strength of the connection was enhanced after the rTMS intervention. After dual-target rTMS, the strength of brain functional network connections between the right PC and right DC brain regions became more comparable to that of normal controls (*p* = 0.450). The changes in brain functional network connection strength before and after rTMS intervention were statistically significantly different (*p* < 0.001). See Figures 3, 4.

Correlation analysis showed that the improvement of MoCA scores after dual-target rTMS stimulation was correlated with the changes in the right PCC to the right DC strength (*R* = 0.605, *p* = 0.022).

## Discussion

Our study found that resting-state functional brain connectivity analysis in MCI patients demonstrated reduced connectivity strength between the right PCC and the right dorsal DC compared to normal controls. After 20 sessions of dual-target rTMS, MoCA scores increased, and various cognitive domains, particularly memory, executive, and attention tests, improved compared to pre-intervention in MCI patients (Table 2). In studies with single-target stimulation of the DLPFC or parietal lobes in MCI patients, the outcome was an improvement in overall cognition or delayed memory function (Cui, et al., 2019; Yuan, et al., 2021). Our protocol improved overall cognitive function compared to the single-target protocol and delayed memory function.

The overall cognitive function was lower in MCI patients than in normal controls, and resting-state functional brain network connectivity analysis revealed abnormal functional connectivity of the right PCC to the right DC in MCI patients (Figures 3, 4). The functional connectivity of the right PCC to the right DC was reduced in MCI patients compared to normal controls. This abnormal decline may be caused by the reduced function of the right PCC with the dorsal DC in the brains of MCI patients. The DC is highly connected to the DLPFC and plays an essential role in motor and cognitive functions, involved in spatial working memory and deductive reasoning (Huang, et al., 2017). The PCC is a vital hub of the default network and belongs to the classical Papez memory circuit, which involves episodic memory and spontaneous cognitive processes (Kvavilashvili, et al., 2020). Previous studies have found that reduced functional connectivity and metabolism of PCC locations are partially responsible for early episodic memory deficits in AD and amnestic mild cognitive impairment (aMCI) (Kvavilashvili, et al., 2020). The abnormalities in PCC may contribute to the decline in cognitive function. After dual-target rTMS intervention, the strength of functional connectivity between the right PCC and the right DC increased and became more comparable to that of normal controls. This result revealed a potential mechanism by which dual-targeting improves cognitive function, possibly by enhancing the functional connectivity between the right PCC and the right DC.

Dual-targeted rTMS significantly improved overall cognitive function, including memory, executive, and attentional functions in MCI patients. This finding is consistent with previous studies reporting that rTMS improves cognitive function in MCI patients (Zhang, et al., 2021). Two locations of our stimulation affected the DMN, a network consisting of the PCC/PCu, medial prefrontal cortex, inferior parietal lobule, lateral temporal cortex, and hippocampal structures (Buckner, et al., 2008). The DMN is associated with higher cognitive functions and is a widely explored brain network in patients with MCI and AD (Krajcovicova, et al., 2014). The DMN is susceptible to transcranial pathology, and evidence suggests that MCI patients often show spontaneous abnormal brain activity in the posterior part of the DMN (Melrose, et al., 2018). Progressive damage to the DMN over time has been observed in MCI patients (Bai, et al., 2011). Unnatural functional connectivity strength within the DMN of MCI patients may be correlated with cellular and structural abnormalities in DMN-related regions (Kvavilashvili, et al., 2020). Dual-target rTMS may enhance the excitability and function of the DMN. Previous research has confirmed that rTMS interferes with local brain network nodes (Eldaief, et al., 2011). Its energy can be transmitted to distant locations *via* synapses, such as to other node locations with spatially specific functional interconnections (Ruff, et al., 2008; Halko, et al., 2014). Dual-targeted rTMS can affect the entire DMN by influencing the PCC and, thus, the DMN. Our study indicates that dual-target rTMS may further enhance the excitability of the default network and improve the overall cognitive and memory functions in MCI patients. Changes in functional connectivity between the right PCC and right DC after stimulation were positively correlated with improvements in MoCA scale scores. It indicates that increasing the strength of the functional network connection between the right PCC and the right DC in MCI patients may be associated with improving cognitive function. These results suggest that dual-target rTMS may be a practical approach to improving cognitive decline in MCI.

Our functional brain network partitioning uses the Brainnetome template, which is more refined than the commonly used AAL partitioning and consists of 210 cortical and 36 subcortical subregions with information on anatomical and functional connectivity (Fan, et al., 2016). Instead of using the traditional subjective banding method, the EEG bands were extracted using the adaptive banding “gedBounds” method. It can reduce the uncertainty and subjectivity of conventional banding, avoid the differences between groups (such as patient groups or genetic backgrounds) and individuals (e.g., age, personality, and like) factors, and contains more detailed and potential information (Cohen, 2021). Unfortunately, the method is currently stable only in the alpha band. Functional neurological analysis can be facilitated *via* future research studies in this field. Advanced algorithms (Li, et al., 2017) may be implemented in the future to improve neurological healthcare services. Functional neurological analysis (Shi, et al., 2019) can be facilitated *via* future research studies in this field.

There are some shortcomings of this study. First, the sample size of the study was small. Second, there was no sham stimulation control group in this trial, which may cause bias. Furthermore, the duration of the rTMS effect was not followed up. Therefore, future studies need to increase the sample size, set up a sham stimulation control group for a randomized controlled trial, and monitor changes in the cognitive status of MCI patients at a regular follow-up to further validate this study’s findings. Future studies could also use MRI navigation techniques for more precise stimulation purposes. The implementation of MRI for neurological analysis is encouraged in future implementations. Implementing EEG and MRI neurological analysis (Zeng, et al., 2016; Li, et al., 2017) is encouraged in future implementations.

In summary, the results of this study suggested that dual-target rTMS can improve cognitive functions, memory, executive functions, and attentional operations in MCI patients. The improvement of these functions may be related to the changes in functional brain connectivity between the right PCC and the right DC in MCI patients. Dual-target rTMS improves functional connectivity between the right PCC and the right DC in MCI patients. This finding will contribute to developing successful TMS treatment protocols for the cognitive rehabilitation of MCI patients.

## Data Availability

The raw data supporting the conclusion of this article will be made available by the authors, without undue reservation.
